# The Role of Environmental Factors in Modulating Immune Responses in Early Life

**DOI:** 10.3389/fimmu.2014.00434

**Published:** 2014-09-12

**Authors:** Duncan M. MacGillivray, Tobias R. Kollmann

**Affiliations:** ^1^Division of Infectious and Immunological Diseases, Department of Paediatrics, Child and Family Research Institute, University of British Columbia, Vancouver, BC, Canada

**Keywords:** evolution, environment, innate immunity, immunologic memory, immune system, pediatrics, ontogeny

## Abstract

The concept of immunological memory stipulates that past exposures shape present immune function. These exposures include not only specific antigens impacting adaptive immune memory but also conserved pathogen or danger associated molecular patterns that mold innate immune responses for prolonged periods of time. It should thus not come as a surprise that there is a vast range of external or environmental factors that impact immunity. The importance of environmental factors modulating immunity is most readily recognized in early life, a period of rapidly changing environments. We here summarize available data on the role of environment shaping immune development and from it derive an overarching hypothesis relating the underlying molecular mechanisms and evolutionary principles involved.

## Introduction

The immune system is an organ that specializes in responding to environmental exposures. Its tasks are to determine friend from foe, innocuous from dangerous, inert from toxic. This highly complex task is most readily recognized during rapid changes of environmental exposures such as those occurring in early life. The purpose of this review is to amalgamate existing data into a cohesive vision on how early life environmental exposures leave a lasting impression on the human immune system, and how this impression can either have beneficial or potentially deleterious effects. This vision incorporates the key principles of the developmental origin of health and diseases (DOHaD) hypothesis, the hygiene hypothesis as well insights from the field of developmental immunotoxicology (DIT) and posits that the sum of these mold immune memory. Immune education includes not only the classical acquired adaptive immune system with the cardinal feature of long-term immune memory but also the more recently described trained memory of the innate immune system. Understanding the environmental engines that drive development of the immune system is not only necessary to address specific pediatric diseases but also to identify the strategies to change trajectories toward long-term, life-long protection from disease.

## Ontogeny of the Immune System

Ontogeny refers to the study of development. As multicellular life evolved so too did cell types with discrete functions. The immune system has evolved to meet a fundamental challenge of multicellular life determining whether exposures are benign or harmful. The seeding and growth of mammalian organs are a highly regulated sequence of events. Periods of rapid development represent windows of vulnerability for dysregulation with subsequent downstream effects that may manifest much later in adult life. Our interest in the immune system is focused upon how it comes to be fully functional, and to try to elucidate how and when aberration can result in disease, and how to best prevent undesirable outcomes. Thus, it is necessary to first understand the earliest origins of immune cells.

Cells making up adaptive and innate arms of the immune system are derived from lymphoid or myeloid progenitors. Hematopoietic stem cells (HSCs) are the canonical precursors of all immune cell lineages and are defined by their capability to replace all blood lineages in lacking recipients (1). HSCs migrate from the yolk sac and aorta-gonad-mesonephros region into the fetal liver, to ultimately reside in the bone marrow in adults where they constantly self-renew and differentiate to replace the rapid turn over of circulating immune cells. In addition, there are multipotential progenitors that can give rise to myeloid and lymphoid cells independent of the yolk sac (2–4). These cells may arise from within the embryo or from extra embryonic sites such as the placenta and vitelline arteries (5). Cell types such as tissue resident macrophages may be derived from monocytes of bone marrow origins or from self-potentiating cells of embryonic yolk sac origin (6). It is yet unknown whether cells with similar roles and phenotypes derived from independent origins have identical functions, programing, and responses to immunological challenges.

Age dependent differences in human immunity are implied by the clinical observation of altered disease susceptibility and substantiated by differences in immune cell activities. Newborns are more susceptible to several diseases when compared to adults; this appears to be at least partially due to a lack of acquired immune memory and differential regulation of innate immune responses (7, 8). This altered immunological priming is not maladaptive, rather, the fetus and neonate are challenged with balancing defense against infection, minimizing potentially harmful inflammation, and mitigating colonization by microbes as it develops and transitions from the relatively protected womb to the external world (9). Over the first few years of life the capacity for the immune system to exert a balanced and rapid response becomes increasingly salient. Though human neonates are capable of mounting adaptive T-cell responses, CD4 T-cell responses are slower to develop and have a predisposition to T helper-2 (Th2) or Th17 responses against extracellular pathogens (10). In contrast, Th1 responses are important for defense against intracellular pathogens and cell mediated immunity, and reach adult levels only after about 2 years of age (10).

The abridged Th1 response is manifest in reduced defense against intracellular pathogens and is influenced in part by different innate cell cytokine responses to pathogen associated molecular patterns (PRRs) that direct subsequent adaptive immune functions (11). These are receptors associated with the innate immune system that recognize conserved regions on broad phyla of organisms and through signaling cascades guide subsequent immune responses. Neonatal innate immune cells including monocytes, plasmacytoid dendritic cells, and conventional dendritic cells produce less interleukin (IL)-12p70 and type I interferon and similar or higher levels of IL-1Beta, IL-6, IL-23, and IL-10 than adult cells when stimulated by the same PRRs (8). In addition to compromised cytokine response, neonatal cells have also been shown to exhibit relatively limited ability to produce multiple cytokines in polyfunctional responses to PRR stimulation. Perinatal anti-inflammatory cytokine production of IL-10 decreases in concert with increasing pro-inflammatory responses (such as tumor necrosis factor alpha and IL-1Beta) over the first few years of life while neonatal antiviral cytokine (type 1) responses already reach adult levels after the first month of life (12). Underlying patterns of immune ontogeny lead to windows of vulnerability to different types of infection during different stages of development (13). While age dependent development of the immune response may also follow genetically encoded programs, increasing evidence from several fields suggest that these changes in immunity are profoundly impacted by external environmental influences. Exemplified by successfully acquired specific and non-specific responses to vaccination, programing early in life represents not only a window of vulnerability but also a window of opportunity for long-term prevention of infectious diseases and maintenance of homeostasis and health.

## DOHaD and Early Life Programing

The developmental origins of health and disease (DOHaD) hypothesis have been gradually increasing in popularity since the 1980s. Conceptually, DOHaD posits that presentation of adulthood disease can be traced back to childhood, perinatal, or *in utero* exposures. Observations that nutritional status during gestation can influence organogenesis and metabolism in later adulthood lead Dr. David Barker to pursue and expand the ideas of DOHaD (14). The current paradigm states that fetal adaptations to intrauterine and maternal conditions during development shape structure and function of organs (14, 15). Alternate development and programing are not maladaptations *per se*, but represent transient responses to environmental demands that confer short-term survival, potentially establishing lasting implications for later health. The immune system represents the prime example of the DOHaD concept, as the immune status is precisely defined by the amalgamation of interactions with the environmental exposures of a given individual. The immune system is the DOHaD organ par excellence and even contains mechanistic parallels in that early life programing directs later immune health through epigenetic modifications, the *modus operandi* of DOHaD.

Epigenetic modifications are defined as mitotically or meiotically heritable changes in accessibility of genes for expression that do not involve a change in DNA sequence (16). There are numerous types of epigenetic modifications including DNA methylation, histone modification, nucleosome position, and non-coding RNA expression (17). These modifications alter gene expression and explain how identical DNA can result in entirely different phenotypes and cell lineages. In humans, environmental exposures can drive epigenetic modification that allow for innate immune cell programing as demonstrated by examples from trained immunity (18). Epigenetic programing is also an important factor in adaptive immune T-cell activation (19) and memory response (20). Cell ontogeny is dependent on epigenetics, and development represents a time where immune priming may be taking place with lasting implications for subsequent health and disease.

## Developmental Immunotoxicology

Numerous environmental factors can influence immune activity, and the field of developmental immunotoxicology (DIT) has arisen to assess how chemical, biological, physical, or physiological factors alter the development of the immune system. Immunotoxic substances have traditionally been identified based on observation of pathology in adults, and may miss potential harm during development. Only now are we beginning to investigate how some exposures may affect pediatric populations and human development (21). Following selective examples of naturally occurring and synthetic compounds illustrate how immunotoxic exposures can influence the immune system.

Depending on concentration naturally occurring heavy metals (e.g., Cd, Hg, Pb) can be either immunopotentiating or immunosuppressive (22). Heavy metals are capable of crossing the placenta; the effects of which depend on the dose, length, and timing of exposure (23, 24). Cadmium exposure demonstrates dose-dependent suppression of circulating IgG in children and is associated with adult diabetes and impaired fasting glucose (25, 26). *In utero* exposure to methylmercury can decrease T-cell functionality, alter circulating immunoglobulin levels and has been implicated with low birth weight and preterm birth (27–29). Neonatal immunotoxicity can occur well below adult safety thresholds (30). Arsenic exposure may lead to direct effects on immunity through epigenetic programing involving leukocytes and metabolic physiology (31–36).

The immune system now encounters synthetic compounds absent from our evolutionary history. We know relatively little about the lifelong effects of synthetic compounds and they have only recently been studied for early life exposures. Three major classes of synthetic compounds include tetrachlorodibenzo-*o*-dioxins (TCDDs), perfluorinate compounds (PFCs), and polychlorinated biphenyls (PCBs); all of which are capable of crossing the placenta and the latter two have been detected in breast milk (37–40). Effects on immune function are compound dependent. TCDDs have been associated with hypothyroidism, inhibition of thymocyte maturation, reduced MHC II expression, altered T-cell differentiation, and decreased blood thyroid stimulating hormone (39, 41, 42). PFCs are known to suppress innate immune cytokine responses (43) and appear to lower antibody response to some childhood immunizations (44). PCB exposure has been linked to neonatal thymus size (45), alteration of childhood adaptive immune cell populations, impaired vaccine responses (46), and increases in childhood middle ear infections (47, 48). Synthetic compounds may have long-term effects due to bioaccumulation in tissues with high fat content or relatively long half-lives (averaging around 10 years for PCBs) and slow clearance (37, 49).

Risk of exposure to immunotoxic substances can be mediated by cultural predilections, including diet, alcohol consumption, and smoking of cigarettes. Alcohol use during pregnancy can result in fetal alcohol spectrum disorders (FASD), extensively reviewed elsewhere (50). Briefly, children with FASD have modulated innate and adaptive immunity (51), and maternal alcohol abuse has been associated with infection risk in newborns (51, 52); long-term effects may be potentiated through epigenetic modifications (53–55). Early life exposure to cigarette smoke has been linked to increased respiratory and ear infections (56, 57), altered innate cytokine production (58), and changes in adaptive cell populations (59). Removing or mitigating cultural sources of immunotoxic compound exposure may indeed be attainable but will require multidisciplinary efforts and community engagement.

The field of DIT is demanding increased immune-surveillance in developing subjects and shows promise for explaining some underlying risk of infectious and non-communicable global disease burden. There is limited and incomplete knowledge about the developmental immunotoxicology of many compounds (both synthetic and natural) but there is compelling evidence that these substances can subtly and dramatically alter human immune systems. It has been suggested that even relatively mild or seemingly benign alteration of immune response may set the stage for disease (60). Factors such as lead, methylmercury, TCDD, and tobacco smoke are considered causal for modifying sub-clinical immune dysfunction that compounded with infection can trigger disease states (61). Immunotoxicological effects can take place in the womb, and more thorough understanding of the maternal environment is required to further explore immune programing at this stage.

## Maternal Environment

Pregnancy impacts both neonatal and maternal immune status. Half of neonatal genetic material is paternally derived and can be targeted by maternal immune responses as non-self, resulting in targeted rejection of the developing neonate. Extensive research has revealed that interaction between maternal and fetal tissues is mitigated by local immune evasion as well as maternal immune modulation, though exactly how is not yet fully understood (62). For instance, the syncytiotrophoblasts that make up the majority of the maternal-fetal interface express alternate forms of major histocompatibility complexes (MHC) that may help enable fetal evasion of maternal immune response (63–65). These cells, and the maternal uterine mucosa, produce indoleamine 2,3 dioxygenase in response to interferon-gamma that suppresses adaptive T-cell proliferation (66). Potential adaptive responses may be further abrogated during pregnancy by an expansion of T-regulatory cell subsets. In murine models, T-regulatory cell expansion has been associated with increased susceptibility to some infections. Susceptibility to pathogens was reduced with removal of T-regulatory cell populations; however, this also triggered maternal immune activity and fetal resorption (67). Pregnancy also changes levels of circulating hormones such as estradiol, progesterone, and estriol that have pleiotropic effects on both adaptive and innate immune cells (68). Immune modulatory effects of pregnancy associated hormones may partially explain observed decreases in severity of maternal inflammatory disease, such as rheumatoid arthritis (69, 70), and increased susceptibility to some infections (71, 72). Maternal immune activity is altered during pregnancy both in the womb and systemically. Altered maternal immune status during the perinatal period meets the demands of pregnancy but also can have implications for infectious diseases.

## Disease Susceptibility and Severity Associated with Pregnancy

There is clinical evidence for maternal immune modulation given that pregnant women have different susceptibility and severity to certain infectious diseases when compared to the general population (71). While pregnant, women are more susceptible to malaria, listeriosis, and human immunodeficiency virus (HIV) type 1 infection (72). Evidence from malaria research suggests that altered susceptibility to disease from certain pathogens is likely due to the appearance of newly derived fetal tissues, such as the placenta. In malaria endemic regions with high rates of transmission, women are generally asymptomatic, however, during pregnancy even previously asymptomatic women may present with disease. Acute disease state appears to be related to variable surface antigens generated by the parasite that results in sequestration of infected erythrocyte at the placenta (73). Accumulation of infected erythrocytes results in local inflammation associated with pathogenesis and adverse pregnancy outcomes (74). During pregnancy, women also suffer more severe infections from influenza, hepatitis E virus (HEV), herpes simplex virus (HSV) (72), and with more limited evidence to measles, smallpox, and varicella (71). Decreased proportions of circulating maternal T, B, and NK cells may represent weakened responses against intracellular pathogens that explain increased disease severity to some infections (75).

Maternal infection status has implications for fetal immune development. Diverse pathogens are capable of infecting the developing fetus either via maternal circulation and the placenta or through the uterine tract (76). Chorioamnionitis can lead to preterm birth and associated morbidity and mortality (77). Beyond direct transmission or infection of the neonate, *in utero* exposure to inflammation, soluble factors, and antigen can alter neonatal immune status (78). Maternal infection can have lasting implications for the neonatal immunity both for subsequent infection with the same agents, or in more general immunomodulatory ways that changes risk of other infections (79).

## Maternal Infection Impacts Neonate Immune Programing

Maternal infection during gestation has implications for neonatal immunology. Effects can be specific to the infectious agent or general modulation of the host immune system. Infection with helminths such as *Wuchereria bancrofti* (80–82) and *Ascaris lumbricoides* (83, 84) results in increased risk of post-partum infection in early life. Active helminth infection also has non-specific general effects on host immunity that enable continued parasitic evasion of host defenses. Human helminth infections are associated with skewing of the host immune response to a Th2 predisposition (85), chronic immune activation, hyporesponsiveness, and immune anergy (86). This immune modulation can impair response to and efficacy of some vaccines (87–90). During pregnancy these off target effects can increase mother-to-child transmission of HIV (91) and alters transplacental transfer of circulating antigen specific antibody to other infectious agents, such as TB that could interfere with vaccination (92). Thus, exposure to helminths *in utero* can result in specific and non-specific immune programing changing the risk of perinatal infections and vaccine responses.

## *In utero* Exposure to HIV Modulates Offspring Immunity

Maternal infection can also directly expose the fetus to pathogen that may cross the placenta, be transmitted during birth, or by contact early in life. HIV is a well characterized infectious disease with profound impacts on the immune system. The use of anti-retroviral drug regimes can increase quantity and quality of life for infected individuals, and perinatal treatment can block the majority of mother to child transmission of the virus. However, HIV-exposed uninfected (HEU) children have increased morbidity and morality compared to unexposed children (93, 94). HEU children have been shown to display altered innate cytokine responses early in life (95, 96), impaired T-cell proliferation, reduced cytokine polyfunctionality, and have reported higher and lower responses to vaccination (97, 98).

Altered neonatal immune status may be due to transplacental transmission of soluble immune factors, exposure to viral antigen, or reduction of breastfeeding by HIV+ mothers. Maternal burden of infection may have significant implications for neonatal immune ontogeny; high maternal viremia has been associated with significantly lower CD4+ T-cell count in uninfected progeny (99). Transmission of viral antigens may also be influencing neonatal immune development as a third of HEU children appear to have detectable virus specific responses (100). Breastfeeding can result in vertical transmission of HIV and interfere with vaccine responses; however, avoidance or early cessation of breastfeeding has been shown to be detrimental for infant health outcomes (94, 101–103). The World Health Organization currently recommends for breast feeding as benefits appear to outweigh risks of vertical HIV transmission (104). The lifelong implications of *in utero* exposure to HIV are not currently known but will gradually unfold as this population increases in size and maturity.

## Breast Feeding

Breast feeding provides not only necessary caloric and nutritional provisions for the growing neonate but also important factors for immune development. Human milk contains proteins that aid digestion, have antimicrobial activities, and act as sources of amino acids for the developing neonate. Bile salt-stimulated lipase aid lipid digestion, alpha amylase may promote complex carbohydrate metabolism, beta casein increases the bioavailability of divalent cations such as calcium and zinc, and lactoferrin facilitates uptake of iron, stimulates gut cytokine production and release, and may have antimicrobial effects (105). These and other factors suggest that colostrum and breast milk are important for neonatal immune ontogeny. Human milk contains numerous immune modulatory proteins such as immunoglobulins, lysozyme that degrades gram positive bacterial outer cell walls, and kappa-casein that blocks pathogen binding to the gastric mucosa (105). Soluble and cell secreted cytokines including IL-1Beta, IL-6, IL-8, IL-10, tumor necrosis factor alpha, granulocyte- and macrophage-colony stimulating factor (GM-CSF) are present in human breast milk (106). The first meal of a breastfeeding neonate is generally colostrum that contains more immune factors that regular breast milk, including maternal immune cells such as neutrophiles, macrophages, B, and T-cells (107). The direct role of maternal soluble factors and immune cells consumed by the rapidly developing neonate is unknown, yet, it is tempting to speculate that these factors aid in the establishment and development of microbial communities in the infant gut, while promoting defense against harmful pathogens (108). Regardless of exact immunological mechanisms, beneficial clinical effects from breastfeeding have been reported for both children and mothers.

Systematic review and meta-analysis of breastfeeding research in developed countries has shown that a history of breastfeeding is associated with a reduced risk of acute otitis media, non-specific gastroenteritis, sever lower respiratory tract infections, atopic dermatitis, asthma, obesity, type 1 diabetes, childhood leukemia, sudden infant death syndrome, and necrotizing enterocolitis (109). Beneficial maternal outcomes were also associated with lactation, and reduction of risk for development of type 2 diabetes, breast cancer, and ovarian cancer. Premature cessation of breastfeeding or not breastfeeding was associated with an increased risk for post-partum depression (109). It is noteworthy that this analysis was based on observational studies and does not imply causality. Breastfeeding is important for nutrition and education of the neonatal immune system.

## Perinatal Nutritional Impact

The relationship between nutritional status and immunological competence is a matter of debate. Nutritional deprivation has likely been common throughout our evolution history, thus, selective processes hypothetically favor resilient immune systems that are not impaired by transient episodes of malnutrition (110). However, our current population density, global interdependency, and modern lifestyles represent a mismatch between the selective pressures historically acting on our immune systems and the exposures we face today. In addition, our concern is with promoting immune health for life, which does not equate with the role of diet and immunity required for maximum reproductive fitness. High adherence to the Mediterranean diet during the perinatal period is protective against wheeze and atopy in children (111), demonstrating that maternal diet may be of import not only for the specific caloric and nutritional need of the developing fetus but also for offspring immune modulation (112). Mechanistically neonatal immune programing can take place *in utero* given that food and environment antigen specific T-cell responses are detectable in virtually all neonatal cord blood samples (113). Nutrition may be the source of antigens to which the immune system must become tolerant, and additionally provide factors that themselves modulate immune activity (113). The interrelationship between nutrition and immunity is highly complex; however, there are known nutritional deficiencies that can influence immune activity early in life though these may indeed be transient effects and not lead to lifelong disease susceptibility.

## Micronutrients

Hallmarks of malnutrition include micronutrient limitations and protein-calorie macronutrient deficiencies. Full reviews on micronutrient malnutrition and immunity can be found elsewhere (114–116) and only select micronutrients (selenium, zinc, and vitamin A) known to modulate immunity are highlighted here. Limited maternal dietary selenium also restricts transmission to the neonate that has been reported to result in impaired *in vitro* activation of thymocytes, and decreased proportions of circulating adaptive immune cells in the neonate (117). Zinc deficiency has been associated with impaired growth and immune cell functions (118) and reprograming of the immune system from adaptive to more innate immune responses (119). Randomized controlled trials of zinc supplementation in small for gestational age term infants have resulted in decreases in diarrhea, pneumonia infection, and may reduce overall mortality in some settings (120). Vitamin A is crucial for integrity of barriers, lymphocyte proliferation, and cytotoxic T-cell activity, deficiency reduces the number of circulating immune cells and complement proteins (121). Broad supplementation of micronutrients may not reduce undesirable outcomes, because nutrient-nutrient interactions may increase or decrease availability of other immune modulatory nutrients altering nutritional homeostasis (121). Explicit deficit may not temper immunity but rather a dietary imbalance could be the culprit. Micronutrient deficiencies are plausible modulators of immunity, though the immune modulation based on micronutrient deficiency appears to be transient and in that subsequent supplementation can rescue immune system functionality.

## Macronutrients

Study of human macronutrient deficiencies primarily arises from tragic natural experiments, wherein populations suffer acute or seasonal periods of starvation. The immune system appears to be intrinsically tied to metabolic functions, and depending on timing of macronutrient deficiency may have long-term implications due to lasting epigenetic modifications (122). Study of immune outcome in such settings is intrinsically difficult, and results are primarily based upon retrospective analysis of records rather than controlled experimental settings.

An incredibly thorough retrospective analysis of perinatal acute famine comes from Holland during WWII, during which comprehensive records were kept about famine exposure, pregnancy, and the development of the newborns (123). *In utero* exposure to famine resulted in lower birth weights, later increased rates of mental illness (schizophrenia and antisocial personality disorder), congenital neural defects, obstructive airway disease, coronary heart disease, altered renal function, fivefold increases in breast cancer risk among women and reduced glucose and insulin tolerance (124). Famine exposure effects were not limited to the first generation, but were carried forward into the subsequent generation. Adult offspring born to prenatally undernourished fathers (but not mothers) were on average 5 kg heavier than peers born to unexposed fathers (125). Lasting effects on immunity are implied by heritable metabolic differences, as obesity has been associated with altered baseline inflammation (126). Additionally, records from a Swedish community have revealed that grand-paternal exposure to poor food availability during his prepubescent and slow growth period resulted in a fourfold increase in grandchild risk for diabetes, however, no underlying mechanism explaining the process was described (127). Thus, even relatively transient exposures to starvation may have intergenerational implications for metabolism, and by proxy, immune status.

Other studies focused on primary immune outcomes and nutrient deficit have produced incongruent results. In the Philippines, 14–15 year olds born with small for gestational age birth (related to intrauterine growth restriction) and lasting nutritional deficit have a lower probability of responding to typhoid vaccines than their peers (128). In contrast, assessment of immune implications from perinatal (seasonal) nutritional deprivation in the Gambia later in life (18–24-year-old men) revealed no lasting effect on the proportion of memory and naive T-cells (129). The timing, nature, and duration of perinatal nutritional deficit impacts human immune development in different ways; however, these effects are additionally confounded by local genetics, toxin exposures, cultural practices, and exposure to both inert and infectious organisms.

## Hygiene Hypothesis and Microbial Exposures

The hygiene hypothesis has expanded from initial observations of allergy and social position to include assessment of observed increases in inflammatory diseases, atopy, and allergy inversely correlated with risk of infectious disease and parasite burden (130). During pregnancy the maternal compartment provides nutrition and immune defenses for the neonate and influences microbial colonization. Maternal involvement in microbial colonization may be from *in utero* exposure, acquisition during childbirth, and transmission through breast milk.

There is ample evidence suggesting that the womb and developing fetus are not sterile, thus, it may be the case that neonatal tolerance to certain microbes may begin before birth. Clinical evidence from perinatal infections implies that the womb is not perfectly sterile, and bacteria may invade the uterus from the abdominal cavity, blood, or the cervix (131). Aside from clinical description of pathology, the presence of bacterial DNA in the placenta of healthy vaginal and caesarian births has been reported (131, 132). Bacteria have been detected in umbilical cord blood from elective cesarean section, in the meconium of healthy term births from mothers who had not had probiotic supplementation who had not yet been breastfed, and even in the amniotic fluid of a murine model (133). While the womb may not be sterile and be the stage for limited *in utero* microbial exposures, the vast majority and continued interaction with microbes takes place post-partum.

## Mode of Delivery

As the neonate transitions from the intrauterine environment into the external environment rapid colonization takes place, influenced by mode of birth. Cesarean and vaginal birth result in different microbes initially colonizing the neonate, either reflecting maternal skin and local surface or vaginal microbes, respectively (134, 135). Clinically, children born by cesarean delivery are more likely to suffer from rhinitis, asthma, type 1 diabetes, and celiac disease (136, 137). Vaginal delivery promotes production of numerous cytokines and associated soluble receptors (IL-6, IL-1Beta, sIL-2R, sIL4R, interferon-gamma, and tumor necrosis factor alpha) in both maternal serum and neonatal umbilical cord blood, which are related to immune activation (138). The promotion of these cytokines supports the notion that vaginal birth activates both maternal and neonatal immune system in a pro-inflammatory manner. This response is likely important for initial colonization of the neonate by microbes, and increase in cesarean delivery associated immune dysfunction may be due to altered microbial exposures and baseline inflammation status of both neonate and maternal systems. Post-partum, neonatal gut microbiome composition is likely continually influenced by breast milk that according to culture dependent, independent, and metagenomic methods harbors a diverse community of bacteria (139, 140).

## Microbiota, Disease, and Trained Immunity

Continued interaction with microbes that live upon and within us represents a most intimate environmental exposure and challenge for the immune system. The microbiota colonizing human gut, skin, and mucosal membranes are integral for energy harvest from food sources (141), metabolism (142, 143), and are implied in the education of the immune system (144, 145). Microbes and microbial communities have been implicated in a variety of diseases that include immune involvement including severe malnutrition (146), obesity (142, 143, 147), chronic obstructive pulmonary disorder (148), and irritable bowl disease (149). Animal models reveal that the gut microbiota directly interact with immune activity by changing the proportion of gut mRNA for chemokines, receptors, and FoxP3 (associated with regulatory T-cells) (150). More comprehensive reviews on the impact of human microbiota in early life are provided elsewhere (144, 151). To date, there has been limited research assessing the role of eukaryotes in microbial communities, further research of which are required to fully describe complex gut ecosystems in order to understand the role of the microbiome in health and disease. The human gut microbiome is acquired from local environments and is variable depending on numerous environmental influences, including geography, diet, and lifestyle (152).

The human microbiota appears to have some implications in human health and disease, likely through participation in immunological education. Early life microbial exposure may be exceedingly important within the context of trained innate immunity. In this process, independent of adaptive immune mechanisms primary exposure to pathogen can alter innate immune cell programed responses to subsequent re-exposure. Depending on the stimuli, exposure can result in innate cell training with resulting heightened or attenuated cytokine responses, lasting programing occurs through epigenetic histone modification (153). Both murine and human studies indicate innate immune system responses are altered by repeated exposure to the same pathogen (154). Innate immune cells can be trained by prior exposures, and can influence the success of vaccines (18). It is of critical importance to better understand how microbial exposure can alter both trained and adaptive arms of the immune system to promote adequate immune education for life-long health.

## Global Variation in Early Life Immunity

Humans have incredibly plastic immune responses to the myriad of toxic, infectious, nutritional, and microbial exposures encountered throughout life. With added genetic variability, it is no wonder that promoting global health has been an ongoing struggle. Age-related patterns in innate immunity development vary depending on the area assessed (155, 156). In efforts to describe global innate immunity Smolen et al. enrolled subjects from four continents and assessed pattern recognition receptor mediated responses. Despite environmental variation between continents toll-like receptor mediated cytokine production was remarkable similar in most sites, with only South African subjects appearing as obvious outliers, though nuanced differences were present between all populations (157). Whether they may be drastic or nuanced differences, understanding how populations response to immune stimuli may be of critical importance for explaining global variation in vaccine efficacy. Global and regional assessment of local immune responses will be fundamental for developing new and optimizing current vaccines.

## Variable Vaccine Response

Even when cold chain and distribution requirements are adequately met vaccines are not ubiquitously successful. One of the best studied and most globally relevant examples of geographic variation in immune system function are the response to vaccination with bacille Calmette-Guerin (BCG) against tuberculosis (TB). Widely used with variable outcomes, a comprehensive meta-analysis of BCG has shown that it reduces tuberculosis risk by 50% (158), but provides no protection in some parts of the world (159). A review of BCG trials found latitude to be strongly associated with protection, explaining 41% of the variance between studies (159). A more recent meta-analysis revealed that protection was higher in trials further from the equator, in areas with lower risk of diagnostic detection bias, and when studies address potential confounding of latent mycobacterial exposure (160).

There are many hypotheses for the variable efficacy of BCG against pulmonary tuberculosis, including vaccine substrain over attenuation, inadequate dosage, interference by prior exposure to *Mycobacterium*, host genetics, and geographic differences in clinical isolates (161). Northern Malawi is an area where BCG does not appear to have substantial protective effects, and prior environmental exposure to mycobacterial antigens appears to prevent replication of BCG within the host (162). Additional research assessing cytokine production induced by vaccination showed UK subjects (who are protected by vaccine) exhibited Th1 associated cytokines, whereas Malawian subjects with poor protection from vaccine had Th2 and IL-17 biased responses (163).

Differences in BCG strains have been well assessed and have been found to lead to differential gene regulation and efficacy, but no one strain appears to be consistently superior in all locations and for both pulmonary and disseminated TB (164). Restimulation of peripheral mononuclear blood cells from Mexican neonates vaccinated with different BCG stains displayed significantly different levels of interferon-gamma, IL-12B, IL27, IL-1, IL-6, and IL-24 mRNA (165). Ultimately, any and all the hypotheses likely have some credence, and determining the best course of action will have to consider local genetics and environmental exposures to optimize vaccine efficacy and disease prevention. Unfortunately, this means no single vaccine is likely to confer protection to all of humanity. However, assessment of local immune responses to stimuli may enable derivation of customized adjuvants to adequately activate the immune system to confer lasting protection.

Despite contrary results for the protective effects of BCG against tuberculosis, vaccination is recommended to continue due to reduced infection from other mycobacteria as well as astonishing non-specific effects that have been recently noted in a randomized clinical trial in premature births in Guinea-Bissau. Overall child mortality was reduced by over 50% when low birth-weight children who received BCG earlier than currently recommended (166). It is important to note that though BCG vaccination may have beneficial non-specific effects, it can also modulate immune responses to subsequent vaccination (167). Non-specific effects of vaccines are not limited to BCG, but have also been reported for measles, and DTP (diphtheria, pertussis, and tetanus) vaccines; it has been suggested that optimization of immune scheduling in areas with high infectious disease burden could reduce child mortality by 30% (168). Optimization of existing vaccine scheduling may seem to be low hanging fruit, but will require thorough analysis of local immune development with consideration of both genetic and environmental factors.

## Seasonality

Though an individual may remain geographically constrained their environmental exposures may vary dramatically depending on seasonal. Changing temperature, rainfall, exposure to elements, food availability, diet, and exposure to infectious agents may be compounded or alleviated by local cultural practice. Perturbation of immune function early in life may have important short-term impacts as both birth season and nutritional status have been associated with decreased humoral response to pertussis vaccination (169). Birth during the wet season in West Africa increases the proportion of T-cells in the CD8+ compartment, decreasing the CD4+:CD8+ ratio (170, 171). In rural Gambia, season of birth has been associated with infection related adult mortality and was postulated to be due to perinatal nutritional status or pathogen exposure during early immune programing (172). However, no association between seasonality and immune status was detected in a subsequent study of youth (173) or young adults (129). Independent of nutrition or pathogen exposure, seasonality can also change diurnal exposure to sunlight and levels of circulating vitamin D. In the United Kingdom, vitamin D status during gestation and birth month has been associated with diseases with immune involvement, including rheumatoid arthritis, irritable bowel diseases, and multiple sclerosis (174). Assessment of seasonality, vitamin D, and infectious disease has been further explored with regards to TB in South Africa, revealing a reciprocal seasonal variation in serum 25(OH)D concentration and TB notification (175). Seasonality is a multifactorial parameter to be considered for studies of human immunity, though evidence for long-term immune programing is limited.

## Conclusion

Numerous environmental factors can modulate human immunity early in life. These range from abiotic chemicals exposures and nutritional status to biotic insult from infectious diseases and with microbial or parasitic colonization. Early life represents windows of both vulnerability and opportunity that impact the developing immune system. The cases above demonstrate that there can be short-term and lasting implications for lifelong health based on pre- and perinatal environmental exposures. These include potential inherited programing (genetic and epigenetic), derailed development due to altered metabolism (nutrition and toxicology), and inappropriate immune system decision-making (for both adaptive and innate arms).

A living world is defined by change, in terms of both environment and human populations. It is fitting that the immune system is in a constant state of flux to adapt to local constraints and conditions. Though selective pressures of environments have shaped the evolution of our immune systems, in recent years we have rapidly altered our lifestyle and environments. As a result, today we live in a world foreign to our ancestors. Given the inherent plasticity of the immune system, it follows that with exposure to entirely different organisms and chemicals than those we coexisted with for the last millenia can result in undesirable outcomes. Through medical interventions we are also living much longer lives, beyond the threshold of selective pressure. In order to promote immune-mediated health for life, we must consider the importance of environmental exposures for immune programing, and learn how to direct the developing immune system to optimize health outcomes. For example, the result of perinatal toxic exposures highlight the need to include assessment of the developing immune system for modulation in animal models beyond short-term adult toxicity, and include multi-generational monitoring. This also suggests surveillance of human populations need to be conducted for longer periods of time, to determine whether chemical exposures are hazardous for immune health in different physiologically relevant combinations and over time. And while we only recently have begun to understand that the microbiome is likely centrally involved in a number of chronic diseases, such profound and wide-spread impact highlights the power of microbial exposure in early life to modulate developmental trajectories. For example, it appears possible to reduce global morbidity and mortality with targeted early life probiotic interventions, as has been recently suggested in preliminary results from an active yogurt culture study in India (176).

Bass Becking and Beijerinck once stated that “everything is everywhere; but the environment selects” (177). We would like to add to this phrase “and we adapt.” To enable continued survival the human immune system must be especially malleable early in life, to respond to rapidly changing requirements between prenatal and postnatal life. Adaptability is the hallmark of both innate and adaptive immunity and through immune memory, can direct life-long immune-mediated health. Though there remains much to be done to determine how to best to direct the immune system toward optimal long-term health, we now have gathered a critical mass of insight into the mechanisms underlying immune programing in early life and with that for the first time are presented with the unprecedented opportunity to become stewards of not only prevention of disease but also promotion of life-long health.

## Conflict of Interest Statement

The authors declare that the research was conducted in the absence of any commercial or financial relationships that could be construed as a potential conflict of interest.
